# Incorporating the Malnutrition Screening Tool and the Malnutrition Universal Screening Tool in Rehabilitation Practice: Comparison With the Nutrition Risk Screening 2002

**DOI:** 10.1002/fsn3.4676

**Published:** 2025-01-09

**Authors:** Kübra Tel Adigüzel, Hatice Aybüke Çalişkan, Fatma Berna Işik, Hilal Çaybaşi Erdoğan, Sena Akşit, Suna Mansiz, Emre Adigüzel, Evren Yaşar

**Affiliations:** ^1^ Gülhane Health Sciences Faculty, Department of Nutrition and Dietetics University of Health Sciences Turkey Ankara Turkey; ^2^ Ankara Bilkent City Hospital, Physical Medicine and Rehabilitation Hospital University of Health Sciences Turkey Ankara Turkey; ^3^ Gülhane School of Medicine, Department of Physical Medicine and Rehabilitation University of Health Sciences Turkey Ankara Turkey; ^4^ Yozgat Bozok University Yozgat Turkey

**Keywords:** malnutrition, malnutrition risk, rehabilitation

## Abstract

To demonstrate the prevalence of malnutrition risk in a specific rehabilitation setting. The secondary aim of the study was to compare Malnutrition Screening Tool (MST) and Malnutrition Universal Screening Tool (MUST) with Nutritional Risk Screening‐2002 (NRS‐2002). Patients diagnosed with stroke, anoxic brain injury, spinal cord injury, multiple sclerosis, arthritis, neuromuscular diseases, Parkinson's disease, and lymphedema who were admitted to a rehabilitation hospital were included. NRS‐2002, MST, and MUST were used to assess malnutrition risk. Body weight (kg), height (cm), and mid upper arm circumference (cm) were measured. Twenty‐four hours dietary records were obtained. Routine blood test results were recorded from patient files. Five hundred sixteen patients with a mean age of 54.3 ± 18.0 years were included. The most prominent diagnoses were stroke and spinal cord injury. According to NRS‐2002, 71.7% (*n* = 370) of the patients were at low risk, but 28.3% (*n* = 146) of the patients were at high risk. Comparisons between NRS‐2002 and MST showed that these two scales have similar results at classifying patients for malnutrition risk (*p* = 0.154). Comparison between NRS‐2002 and MUST showed significant differences (*p* < 0.001). Both sensitivity and specificity of MST were above 80.0%. Sensitivity of MUST was 78.1% and specificity was 88.1%. Approximately one‐third of the patients were at risk of malnutrition. Specificity and sensitivity of MST and MUST were as high as routinely used scale NRS‐2002, and therefore it can be supposed that, considering the diagnostic groups of the patients, MST and MUST are useful in rehabilitation practice.

## Graphical abstract

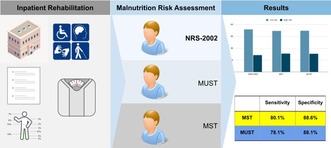

## 1 Introduction

Malnutrition is defined as “a state resulting from lack of intake or uptake of nutrition that leads to altered body composition (decreased fat mass) and body cell mass leading to diminished physical and mental function and impaired clinical outcome from disease” by the European Society for Clinical Nutrition and Metabolism (ESPEN) (Cederholm et al. 2017). Malnutrition is a significant health issue that can stem from primary factors like poverty, leading to food scarcity, or secondary factors, such as illness (Cederholm and Bosaeus 2024). As a result, inadequate nutrition detrimentally influences the diagnosis, prognosis, and progression of both acute and chronic diseases (Serón‐Arbeloa et al. 2022). In order to predict the extent of health burden caused by malnutrition, it is necessary to know how many people or patients this condition affects. Although underdiagnosed, rates of malnutrition in public were reported to be 8.5%–17.0% (Abizanda et al. 2016; Roberts, Collins, and Rattray 2021).

Researches about malnutrition generally focus on acute disease situations, but relatively less attention is given to the prevalence and the effect of malnutrition on rehabilitation patients. In a previous review, malnutrition prevalence in general rehabilitation settings was reported as ranging from 35% to 51% (Di Vincenzo et al. 2023). In a more specific patient group of stroke, 6%–62% of prevalence was found by (Foley et al. 2009). This wide range of prevalence was linked to the different patient characteristics, different assessment tools, and timing of screening (Foley et al. 2009; Di Vincenzo et al. 2023).

In rehabilitation practice, as well as dealing with diseases, due importance is also given to improving the factors that negatively affect the rehabilitation outcomes. Previously, it was shown that malnutrition is associated with longer length of stay and higher mortality (Foley et al. 2009). In addition, it is known that malnutrition negatively affects rehabilitation patients in both short and long term with increased disability and mortality (Huppertz et al. 2022). Examples of these negative effects are delayed and poor wound healing, cognitive and physical impairments, infections, lower quality of life, and pressure ulcers (Di Vincenzo et al. 2023; Charlton et al. 2010; Demarest‐Litchford et al. 2024).

Therefore, it is obvious that interventions to detect and prevent the progression of malnutrition and associated complications will provide positive results on patient outcomes. To adequately perform a nutritional screening in clinical practice, a reasonable first step is to select an appropriate tool. Various screening tools have been developed to assess nutritional status across different populations and settings (Cortes et al. 2020). These tools vary in complexity, specificity, and sensitivity, and their applicability depends on the context in which they are used. Nutrition risk screening‐2002 (NRS‐2002) is the tool which is used routinely for inpatients. Malnutrition Screening Tool (MST) and Malnutrition Universal Screening Tool (MUST) are some of the other tools used for the same purpose (Serón‐Arbeloa et al. 2022; Cortes et al. 2020). In practical use, regarding data collection such as anthropometric measurements, food intake, weight loss, etc., these tools have some disadvantages and also advantages. In the hospital, where the current trial is conducted, NRS‐2002 is routinely used for every admitted patient.

The primary aim of the present study was to demonstrate the prevalence of malnutrition risk in a specific rehabilitation hospital. The secondary aim was to compare MST and MUST with NRS‐2002, regarding sensitivity and specificity. Overall, by identifying and considering the risk of malnutrition definitely, a significant secondary factor that needs to be ameliorated during the rehabilitation process will be improved.

## 2 Materials and Methods

### 2.1 Design and Setting

This single‐center cross‐sectional study was conducted in a tertiary Physical Medicine and Rehabilitation Hospital which consists of twelve inpatient clinics. This hospital accepts patients who were diagnosed with neurological or musculoskeletal diseases from surrounding hospitals within a large medical campus, and in addition, admits patients from its own Physical Medicine and Rehabilitation outpatient clinics. Inpatients of ten clinics were selected: five stroke and acquired brain injury clinics (125 beds), two spinal cord injury rehabilitation clinics (50 beds), one neuromuscular rehabilitation clinic (25 beds), and two general rehabilitation clinics (50 beds). Since pediatric patients were followed in the remaining two clinics, inpatients of these clinics were excluded.

A sample of 597 consecutive patients was evaluated between December 2021 and December 2022. Patients were included if they met the following inclusion criteria: over 18 years of age, have the communication level to obtain information about his/her nutritional status (by himself/herself or caregiver), and volunteer to participate in the study. No exclusion criteria were present.

Diagnoses of these patients were stroke, anoxic brain injury, spinal cord injury, multiple sclerosis, arthritis, neuromuscular diseases, Parkinson's disease, and lymphedema. All patients were diagnosed according to the up‐to‐date criteria by physicians in the clinics.

### 2.2 Anthropometric Measurements

Body weight (kg), height (cm), and mid upper arm circumference (cm) of the participants were measured by an experienced dietitian. Weight of the patients who were able to stand independently was measured by body weight scale (Tanita HD‐366; Tanita, Arlington Heights, IL, USA).

Wheelchair scale was used to measure the body weight of the patients who were not able to stand independently (Chinesport Platform Scale XWU003). The patient was weighed while in a wheelchair. Then, the weight of the wheelchair used by the patient was measured when it was empty and this weight was subtracted from the total weight. These measurements were performed when the patients were hungry and wearing lightweight clothes. Body mass index (BMI) was calculated using the formula body weight/height (kg/m^2^). Mid upper arm circumference was measured at the midpoint between the tip of the shoulder and the tip of the elbow (Pekcan 2008).

### 2.3 Nutritional Assessment

On admission (in the first 24 h period), the nutritional status of the patients was assessed using NRS 2002, MUST, and MST. NRS‐2002, MST, and MUST were delivered by dietitian researchers who were experienced in using these tools. All practitioners were trained before using the tools.

Nutritional Risk Screening‐2002: NRS‐2002 includes weight loss, nutritional intake, and BMI (1–3 points), disease severity score (1–3 points), and age correction in individuals over 70 years of age (+1 point). Patients are classified as not at risk of malnutrition (< 3 points) and at risk of malnutrition (≥ 3 points) (Kondrup et al. 2003).

Malnutrition Screening Tool : It is a screening tool consisting of 3 questions regarding involuntary weight loss, the level of weight loss, and loss of appetite. Patients are classified as having no risk of malnutrition (< 2 points) and having a risk of malnutrition (≥ 2 points) (Ferguson et al. 1999).

Malnutrition Universal Screening Tool : Three independent criteria, body weight, involuntary weight loss, and the presence of acute disease, are examined in the MUST screening tool. Each parameter is scored as 0, 1, or 2, and patients are classified as low risk (0), moderate risk (1), and high risk (≥ 2) (Malnutrition Advisory Group (MAG) 2000).

Twenty‐four‐hour dietary records of the patients were obtained. Volumes and portion sizes were assessed using a food catalog with photographs of 120 different foods (Rakicioglu et al. 2014). To calculate the daily intake of macro‐ and micronutrients, and energy, the BeBiSpro software version 7.2 (Stuttgart, Germany; 2010) was used (Bebis, (Beslenme Bilgi Sistemi), Nutrition Data Base Software 2004).

### 2.4 Biochemical Analysis

Total protein, albumin, total cholesterol, low density lipoprotein, high density lipoprotein, triglyceride, C‐reactive protein, and hemoglobin values (which are routinely tested on admission) were recorded from the patient files. All blood samples were collected after 8 hours of fasting, at 07:00–08:00 AM.

### 2.5 Statistical Analysis

Statistical analysis of the current study was done via Statistical Package for the Social Sciences (SPSS) version 22.0 for Macintosh (IBM Corp. Released 2013. IBM SPSS Statistics for Mac, Version 22.0. Armonk, NY: IBM Corp.). Continuous data were shown as mean (standard deviation), and categorical data was shown as number and percentages. Kolmogorov‐Smirnov test was used to examine normal distribution. Categorical data was compared with Chi Square test. One‐way ANOVA test was used for comparisons, and Bonferonni correction was used for within‐group comparisons. The capacity of MST and MUST (compared to NRS‐2002) in predicting the presence of malnutrition was analyzed using ROC (Receiver Operating Characteristics) curve analysis. The sensitivity, specificity, and positive and negative predictive values were presented. To evaluate the area under the curve, a 5% type‐1 error level was used to accept a statistically significant value of the test variables. *p* value of < 0.05 was considered significant.

## 3 Results

During the study period, 597 inpatients were evaluated and according to inclusion criteria, 516 of these patients were included. The main characteristics of the patients are shown in Table 1. The most common diagnoses were stroke and spinal cord injury. The majority of the patients with spinal cord injury were paraplegic.

**TABLE 1:** Main characteristics of the patients.

	Mean	SD
Age (years)	54.3	18.0
Disease duration (months)	33.1	63.3
Length of stay at rehabilitation (days)	23.0	21.8
	***n***	**%**
Gender		
Female	181	35.1
Male	335	64.9
Diagnosis		
Stroke	229	44.4
Anoxic brain injury	21	4.1
Spinal cord injury‐paraplegic	145	28.1
Spinal cord injury‐tetraplegic	43	8.3
Multiple sclerosis	17	3.3
Arthritis	15	2.9
Neuromuscular diseases	30	5.8
Parkinson disease	10	1.9
Lymphedema	6	1.2
Comorbid diseases		
Yes	210	40.7
No	306	59.3

Abbreviation: SD, standard deviation.

Risk assessment of the patients according to NRS‐2002, MST, and MUST are shown in Table 2. According to NRS‐2002, 71.7% (*n* = 370) of the patients were at low risk, but 28.3% (*n* = 146) of the patients were at high risk. Comparisons between NRS‐2002 and MST showed that these two scales provided similar results at classifying patients for malnutrition risk (*p* = 0.154). Comparison between NRS‐2002 and MUST showed significant differences (*p* < 0.001). The diagnostic accuracy of MST and MUST compared with NRS‐2002 is shown in Table 3. Both sensitivity and specificity of MST were above 80.0%. Sensitivity of MUST was 78.1% and specificity of MUST was 88.1%.

**TABLE 2:** Malnutrition risk of patients according to NRS‐2002, MST, and MUST.

	NRS‐2002		MST		MUST			
	Low risk	High risk	Low risk	High risk	Low risk	Medium risk	High risk	*p*
All patients	370 (71.7)	146 (28.3)	357 (69.2)	159 (30.8)	358 (69.4)	77 (14.9)	81 (15.7)	0.154^a^,< 0.001^b^
Stroke	139 (60.7)	90 (39.3)	142 (62.0)	87 (38.0)	155 (67.7)	34 (14.8)	40 (17.5)	
Anoxic brain injury	15 (71.4)	6 (28.6)	13 (61.9)	8 (38.1)	14 (66.7)	2 (9.5)	5 (23.8)	
Spinal cord injury‐paraplegic	111 (76.6)	34 (23.4)	105 (72.4)	40 (27.6)	98 (67.6)	28 (19.3)	19 (13.1)	
Spinal cord injury‐tetraplegic	35 (81.4)	8 (18.6)	31 (72.1)	12 (27.9)	28 (65.1)	6 (14.0)	9 (20.9)	
Multiple sclerosis	15 (88.2)	2 (11.8)	14 (82.4)	3 (17.6)	11 (64.7)	3 (17.6)	3 (17.6)	
Arthritis	15 (100.0)	0 (0.0)	14 (93.3)	1 (6.7)	15 (100.0)	0 (0.0)	0 (0.0)	
Neuromuscular diseases	28 (93.3)	2 (6.7)	27 (90.0)	3 (10.0)	25 (83.3)	2 (6.7)	3 (10.0)	
Parkinson disease	6 (60.0)	4 (40.0)	6 (60.0)	4 (40.0)	7 (70.0)	2 (20.0)	1 (10.0)	
Lymphedema	6 (100.0)	0 (0.0)	5 (83.3)	1 (16.7)	5 (83.3)	0 (0.0)	1 (16.7)	

Abbreviations: MST, malnutrition screening tool; MUST, malnutrition universal screening tool. NRS‐2002, nutrition risk scale.

^a^ NRS‐2002 versus MST.

^b^ NRS‐2002 versus MUST.

**TABLE 3:** Diagnostic accuracy of MST and MUST compared with malnutrition risk according to NRS‐2002.

Tool	Sensitivity, %	Specificity, %	PPV, %	NPV, %	AUC
MST	80.1	88.6	73.6	91.9	0.156
MUST	78.1	88.1	72.2	91.1	0.169

Abbreviations: AUC, area under curve; BFA, body mass index for age; HFA, height for age; MST, Malnutrition Screening Tool; MUST, Malnutrition Universal Screening Tool; NPV, positive predictive valueNRS‐2002, Nutrition risk scale‐2002.

In Table 4, anthropometric measurements and biochemical analysis of the patients are shown. According to diagnosis‐related group comparison, MUAC measurement was different between groups (*p* < 0.001). There was no significant difference in body weight, height, and BMI (*p* > 0.05). Paired group comparisons showed that MUAC difference was present between stroke and lymphedema groups (*p* = 0.001), between tetraplegia and lymphedema groups (*p* = 0.001), and between anoxic brain injury and lymphedema groups (*p* = 0.001). Biochemical analysis did not show significant differences between groups (*p* > 0.05).

**TABLE 4:** Anthropometric measurements and biochemical analysis of patients.

	All patients	Stroke	ABI	SCI‐paraplegic	SCI‐tetraplegic	MS	Arthritis	NMD	Parkinson disease	Lymphedema	*p*
	Mean (SD)	Mean (SD)	Mean (SD)	Mean (SD)	Mean (SD)	Mean (SD)	Mean (SD)	Mean (SD)	Mean (SD)	Mean (SD)	
Weight (kg)	72.1 (14.1)	72.4 (13.7)	71.5 (16.4)	73.0 (14.0)	69.4 (13.0)	68.1 (15.5)	70.7 (13.2)	71.7 (15.5)	69.5 (14.0)	84.0 (18.9)	0.396
Height (cm)	168.1 (12.0)	168.1 (9.4)	170.0 (12.1)	169.4 (9.0)	171.0 (8.3)	165.4 (9.1)	164.9 (11.3)	162.5 (31.9)	159.8 (5.5)	163.6 (13.6)	0.015
BMI (kg/m^2^)	25.4 (5.0)	25.5 (4.8)	24.6 (5.5)	25.4 (4.8)	23.6 (4.3)	25.0 (6.0)	25.9 (4.1)	25.2 (4.8)	27.1 (5.3)	32.1 (9.5)	0.017
MUAC (cm)	27.7 (4.0)	27.5 (3.8)^a^	27.1 (3.5)^a^	28.2 (4.0)	26.4 (3.0)^a^	27.7 (4.4)	28.7 (3.0)	26.7 (4.8)	29.1 (3.3)	35.6 (5.5)^b^	< 0.001
Hemoglobin (g/dL)	12.8 (1.7)	12.8 (1.8)	12.7 (2.3)	12.8 (1.8)	12.4 (1.7)	13.3 (1.3)	12.7 (2.3)	13.0 (1.8)	12.4 (1.5)	11.4 (2.5)	0.396
Albumin (g/L)	69.2 (335.9)	55.9 (253.1)	38.8 (5.5)	119.7 (54.6)	38.8 (5.5)	40.2 (2.5)	40.0 (4.2)	39.0 (4.0)	37.5 (6.4)	39.2 (4.8)	0.784
Triglycerides (mg/dL)	143.6 (73.4)	147.7 (71.1)	159.7 (69.6)	135.6 (80.9)	159.7 (69.6)	135.5 (55.6)	164.2 (109.1)	129.7 (59.4)	152.5 (94.1)	104.4 (25.5)	0.498
Total cholesterol (mg/dL)	163.1 (39.9)	162.9 (39.1)	158.6 (42.3)	160.9 (42.7)	164.0 (37.7)	176.7 (45.1)	179.0 (33.0)	167.3 (36.1)	148.1 (35.6)	152.6 (35.5)	0.526
LDL (mg/dL)	102.7 (70.6)	100.5 (67.9)	91.6 (31.7)	108.7 (84.3)	113.3 (97.4)	102.5 (38.9)	95.6 (23.3)	97.1 (29.6)	79.7 (24.2)	91.5 (31.9)	0.854
HDL (mg/dL)	39.8 (11.6)	39.0 (9.6)	38.6 (12.8)	40.2 (13.2)	37.6 (12.8)	44.4 (11.6)	48.6 (15.8)	41.3 (11.8)	38.3 (10.9)	40.2 (10.4)	0.061
C‐reactive protein (mg/dL)	8.1 (25.8)	9.4 (31.5)	5.0 (9.7)	8.8 (23.5)	9.5 (22.2)	2.0 (2.9)	0.1 (0.2)	5.2 (17.9)	0.9 (2.2)	5.3 (10.0)	0.789

*Note:* Different letters (a and b) indicate significant differences between groups.

Abbreviations: ABI, anoxic brain injury; MS, multiple sclerosis; NMD, neuromuscular disorders; SCI, spinal cord injury; SD, standard deviation.

Feeding type and dietary intake of the patients are shown in Table 5. All patients with spinal cord injury, multiple sclerosis, arthritis, Parkinson's disease, and lymphedema were fed orally. 9.2% of patients with stroke, 28.6% of patients with anoxic brain injury, and 3.3% of the patients with neuromuscular disease were fed via percutaneous endoscopic gastrostomy. There was significant difference between patient groups in terms of feeding type (*p* < 0.001). Energy, carbohydrate, protein, fat, monounsaturated fatty acid, polyunsaturated fatty acid, saturated fatty acid, cholesterol, fiber, vitamin A, vitamin C, and vitamin E intake of the patients were similar between groups (*p* > 0.05).

**TABLE 5:** Feeding type and nutritional intakes of the patients.

	All patients	Stroke	ABI	SCI‐paraplegic	SCI‐tetraplegic	MS	Arthritis	NMD	Parkinson disease	Lymphedema	*p*
	*n* (%)	*n* (%)	*n* (%)	*n* (%)	*n* (%)	*n* (%)	*n* (%)	*n* (%)	*n* (%)	*n* (%)	
Oral	486 (94.2)	206 (90.0	15 (71.4)	145 (100.0)	43 (100.0)	17 (100.0)	15 (100.0)	29 (96.7)	10 (100.0)	6 (100.0)	< 0.001
Oral + PEG	2 (0.4)	2 (0.8)	0 (0.0)	0 (0.0)	0 (0.0)	0 (0.0)	0 (0.0)	0 (0.0)	0 (0.0)	0 (0.0)	
PEG	28 (5.4)	21 (9.2)	6 (28.6)	0 (0.0)	0 (0.0)	0 (0.0)	0 (0.0)	1 (3.3)	0 (0.0)	0 (0.0)	
	**Mean (SD)**	**Mean (SD)**	**Mean (SD)**	**Mean (SD)**	**Mean (SD)**	**Mean (SD)**	**Mean (SD)**	**Mean (SD)**	**Mean (SD)**	**Mean (SD)**	
Energy [kcal]	1496.3 (429.1)	1540.30 (399.8)	1609.6 (475.8)	1441.2 (452.9)	1438.6 (505.5)	1516.2 (466.7)	1414.6 (329.7)	1501.9 (415.8)	1364.3 (404.8)	1506.8 (320.5)	0.380
Carbohydrates [g/day]	159.5 (55.5)	162.0 (52.6)	179.9 (67.5)	153.4 (58.3)	163.9 (63.0)	167.1 (52.1)	156.6 (36.5)	158.7 (55.1)	144.3 (39.4)	124.5 (58.5)	0.358
Protein [g/day]	65.3 (22.7)	68.7 (21.0)	64.0 (27.7)	62.8 (24.9)	57.3 (22.9)	65.4 (22.5)	60.8 (13.6)	62.7 (20.9)	63.6 (24.1)	86.3 (12.1)	0.017
Fat [g/day]	63.9 (22.7)	65.9 (21.5)	66.5 (28.9)	62.7 (22.9)	59.3 (24.5)	63.1 (25.1)	58.9 (20.2)	64.0 (25.5)	57.2 (18.7)	71.5 (23.2)	0.582
PUFA [g/day]	22.2 (11.7)	22.9 (9.4)	22.3 (6.9)	21.5 (7.5)	23.6 (28.4)	20.7 (9.7)	19.6 (5.9)	22.7 (11.6)	18.3 (8.3)	23.8 (8.4)	0.843
MUFA [g/day]	9.7 (5.3)	9.8 (5.1)	9.6 (5.2)	9.4 (5.0)	9.8 (6.9)	9.8 (6.0)	8.8 (4.5)	11.3 (5.7)	8.5 (5.9)	13.0 (6.3)	0.614
SFA [g/day]	26.7 (10.2)	27.6 (10.5)	25.9 (12.5)	26.4 (10.0)	24.2 (9.9)	26.5 (8.1)	26.0 (10.4)	27.7 (10.4)	22.9 (9.9)	29.7 (9.1)	0.594
Cholesterol [mg/day]	286.0 (144.1)	294.4 (150.4)	224.5 (142.8)	272.1 (140.9)	268.1 (137.6)	301.4 (136.2)	285.8 (124.3)	292.2 (129.2)	350.5 (133.2)	447.4 (61.7)	0.047
Fiber [g/day]	17.4 (7.9)	18.1 (7.9)	17.8 (7.7)	16.8 (8.2)	17.4 (9.5)	15.5 (4.7)	15.6 (5.5)	17.4 (5.7)	14.9 (6.3)	18.2 (3.8)	0.701

Abbreviations: MUFA, monounsaturated fatty acid; PEG, percutaneous endoscopic gastrostomy; PUFA, polyunsaturated fatty acid; SFA, saturated fatty acid.

## 4 Discussion

The main findings of this study are that 28.3% of patients were at high risk for malnutrition according to NRS‐2002, 30.8% of patients were at high risk for malnutrition according to MST, and 30.6% of patients were at medium and high risk for malnutrition according to MUST. Sensitivity and specificity of MST and MUST were at acceptable rates, compared to NRS‐2002. The aforementioned findings show that malnutrition risk in this rehabilitation setting is higher than public rates and also MST and MUST are valid tools for malnutrition screening in this patient population.

In the literature, there are studies which investigated malnutrition risk prevalence covering all hospitalized rehabilitation patients (Foley et al. 2009; Charlton et al. 2012). In these studies, different risk scales were used and the results obtained resulted in a wide range. Leipold et al. (2018) conducted a study using MST in a community rehabilitation program and reported that 34.0% of participants were identified as malnourished. Hettiarachchi et al. (2021) used MST in geriatric rehabilitation population and malnutrition risk was 41.3%. On the other hand, some researchers conducted studies in unique diagnostic groups. Scrutinio et al. (2020) investigated a stroke population by using Prognostic Nutrition Index and they reported that 24.0% of participants presented malnutrition (a moderate malnutrition of 12.7% and severe malnutrition of 11.5%). In a similar stroke population, Gomes, Emery, and Weekes (2016) reported that 7.0% of patients presented medium risk and 29.0% of patients presented high risk according to MUST. The research results mentioned above show some differences both with the current research and with each other. As it can be evaluated, malnutrition is common in patients who were admitted to rehabilitation hospitals, but according to the tool used, age distribution, diagnosis of the included patients, and disease duration, different malnutrition rates can be obtained.

In order to predict the risk of malnutrition and make clinical decisions accordingly, the first step is to state the risk clearly. Therefore, specificity and sensitivity of the tool to predict malnutrition risk is of great importance. In a previous study, Leipold et al. (2018) reported 72.2% sensitivity and 83.8% specificity of MST when compared to Subjective Global Assessment in a Community Rehabilitation Program. But diagnostic categories of patients were mostly orthopedic and only 11.7% of the patients had neurologic diagnosis in the study of Leipold et al. Sharma et al. (2022) investigated MUST in an elderly patient group and compared it with Subjective Global Assessment; they reported 69.7% sensitivity and 75.8% specificity. Sensitivity and specificity analysis of the current study showed that MST and MUST both have high sensitivity and specificity for malnutrition screening when compared to NRS‐2002.

Another important issue regarding the malnutrition risk assessment tool is whether it is easy to implement. Previously, it was mentioned by Di Vincenzo et al. (2023) that in stroke patients, especially in acute phase, NRS‐2002 and MUST cannot be used easily. Both of these scales require recall of body weight change in the last 3 months, and data collection may not always be available in intensive care units because of cognitive impairment, aphasia, and visual/hearing impairment. In the current study, the majority of stroke patients were at subacute or chronic stages (mean disease duration of the patients was 33.1 months), and such a difficulty was not experienced by researchers.

The scales used in this study have some common aspects and also some differences. The common point of all three scales is to question recent involuntary weight loss. On the other hand, NRS‐2002 and MUST both consider disease severity. For NRS‐2002, the presence of severe disease alone is not sufficient to categorize a patient as at risk for malnutrition. However, in MUST assessment, substantially acute illness (critical illness with swallowing difficulties such as stroke, brain injury, etc.) scores 2 points, and the patient is reported as high risk for malnutrition. Although this situation may suggest that MUST will cause an overestimation of malnutrition, the specificity value over 80.0% indicates that this was not seen in this patient population in which the study was conducted. Another major difference between these scales is that MST doesn't need BMI data. Although some questions emerge about how to assess malnutrition risk without BMI data, both previous studies and the specificity and sensitivity data obtained in the current study have shown that MST is a valid tool in malnutrition screening (Leipold et al. 2018; Wong et al. 2023). Further studies will be valuable to support this finding.

We acknowledge some limitations of our study. First of all, heterogenous patient population was the major limitation. However, sample size was large, and this provided large patient sizes in subgroups of major diseases such as stroke, tetraplegic spinal cord injury, and paraplegic spinal cord injury. Secondly, only MST and MUST were compared with NRS‐2002, but there are other valid tools such as Mini Nutritional Assessment, Subjective Global Assessment, and Short Nutritional Assessment Questionnaire. Because of these severely affected patient groups, other malnutrition screening tools could not be used, and more useful results could have been obtained if comparisons were made with different tools. Additionally, the dietary records of the patients were only reflecting 24 hours. Longer dietary records would provide more accurate results. The major strong side of the current study was the large sample size. In addition, all researchers were trained about NRS‐2002, MST, and MUST.

## 5 Conclusion

The results of the current study showed that approximately one‐third of the patients were at risk of malnutrition. Specificity and sensitivity of MST and MUST were as high as the routinely used scale NRS‐2002, and therefore it can be supposed that, considering the diagnostic groups of the patients, MST and MUST are useful in rehabilitation practice. If the effects of malnutrition on rehabilitation outcomes are taken into account, paying more attention to these screening tools and promoting their use may provide positive results in terms of patient outcomes.

## Author Contributions

**Kübra Tel Adigüzel: Conceptualization (lead), data curation (equal), formal analysis (lead), funding acquisition (equal), investigation (lead), methodology (lead), project administration (lead), resources (lead), supervision (equal), validation (equal), visualization (equal), writing – original draft preparation (equal), writing – review and editing (equal). Hatice Aybüke Çalişkan:** data curation (equal). **Sena Akşit:** data curation (equal). **Hilal Çaybaşi Erdoğan:** data curation (equal). **Fatma Berna Işik:** data curation (equal). **Suna Mansiz:** data curation (equal). **Emre Adigüzel:** formal analysis (equal), investigation (equal), methodology (equal), software (equal), validation (equal), visualization (equal), writing – original draft (equal). **Evren Yaşar:** funding acquisition (equal), supervision (equal), writing – review and editing (equal).

## Conflicts of Interest

The authors declare no conflicts of interest.

## Data Availability

The data that support the findings of this study are available on request from the corresponding author. The data are not publicly available due to privacy or ethical restrictions.
